# UTILIZATION OF LASSO AND POISSON REGRESSION DEALING WITH COUNT VARIABLES IN NURSING HOME RESEARCH

**DOI:** 10.1093/geroni/igad104.2916

**Published:** 2023-12-21

**Authors:** Juh Shin, Eun Jeong Min, Sun Ok Jung, Jung Eun Kim

**Affiliations:** George Washington University, Washington, District of Columbia, United States; The Catholic University of Korea, Seoul, Republic of Korea; Ewha Womans University, Seoul, Republic of Korea; California State University, Los Angeles, Los Angeles, California, United States

## Abstract

Regression has been used for decades; thus, more empirical studies are necessary to choose appropriate regression methods, as traditional regression has many drawbacks and is limited in analysis and synthesis with large numbers of covariates. This study aims to investigate factors related to pressure ulcers in nursing homes using three regressions: least absolute shrinkage and selection operator (LASSO) Poisson, LASSO linear, and LASSO logistic regression. We represent how each model’s estimating, inferencing, and performance measures work. The datasets accrued from 51 nursing homes that operated between 2021 and 2022. A total of 35 independent variables included organizational and nurse staffing variables. The variable of interest was the number of residents in each nursing home who had an occurrence of pressure ulcers within the last 3 months. The significant factors were different; turnover of Certified Nurse Aides and government incentives of more RNs were significant in the LASSO Poison regression, no factors were significant in the LASSO Linear regression, while and the percentage of residents who are completely dependent for activities of daily living in LASSO was significant factor in the Logistic regression. Using the coefficients in the Poisson regression, we interpreted that more nursing staff turnover increased the percentage of residents with pressure ulcers by 3.02 times, which would not be possible with linear regression. The Lasso model uses a technique of shrinking coefficient estimates to zero to improve the fit of a model that includes all explanatory variables and improves model interpretability by identifying most important features.

